# Lower serum insulin-like growth factor 2 level in patients with bipolar disorder is associated with the severity of manic symptoms during manic episodes

**DOI:** 10.3389/fpsyt.2024.1354999

**Published:** 2024-03-18

**Authors:** Shi-Yi Ye, Ying Zhao, Zhao-Bo Liu, Cui-Pin Luo, Jian-Wen Xiong, Jin-Qiong Zhan, Yi-Heng Li, Bo Wei, Chun-Nuan Chen, Yuan-Jian Yang

**Affiliations:** ^1^ Department of Psychiatry and Biological Psychiatry Laboratory, Jiangxi Mental Hospital & Affiliated Mental Hospital, Jiangxi Medical College, Nanchang University, Nanchang, Jiangxi, China; ^2^ The 3^rd^ Clinical Medical College, Jiangxi Medical College, Nanchang University, Nanchang, Jiangxi, China; ^3^ Department of Pharmacy, Union Hospital, Tongji Medical College, Huazhong University of Science and Technology, Wuhan, China; ^4^ Department of Psychiatry, Third People’s Hospital of Ji′an City, Ji′an, China; ^5^ Nanchang City Key Laboratory of Biological Psychiatry, Jiangxi Provincial Clinical Research Center on Mental Disorders, Jiangxi Mental Hospital, Nanchang, Jiangxi, China; ^6^ Department of Neurology, The Second Clinical Medical College, The Second Affiliated Hospital, Fujian Medical University, Quanzhou, China

**Keywords:** bipolar disorder, insulin-like growth factor-2, manic symptoms, severity, serum

## Abstract

**Objective:**

Accumulating evidence has indicated that neurodevelopmental defects may underlie the pathophysiology of bipolar disorder (BD). Insulin-like growth factors (IGFs) are a family of neurotrophic factors that are essential for the survival and development of neurons. The present study aims to investigate whether IGF-2 signaling is implicated in the pathophysiological processes of BD.

**Method:**

50 healthy controls and 78 patients with BD, including 23 patients who diagnosed acute depressive episode and 55 patients who diagnosed acute manic episode, were recruited in this study. The 17-item Hamilton Depression Rating Scale (HAMD-17) and the Young Mania Rating Scale (YMRS) were used to assess the severity of the depressive and manic symptoms, respectively. The serum IGF-2 level was determined by an enzyme-linked immunosorbent assay (ELISA). The Kolmogorov-Smirnov and Mann-Whitney U tests were used for between-group comparisons and spearman analysis was used to analyze correlations.

**Results:**

Patients with BD had lower serum IGF-2 levels (66.08 ± 21.22 ng/ml) when compared to healthy controls (88.72 ± 31.55 ng/ml). BD patients were divided into manic episode and depressive episode subgroups. We found that serum IGF-2 levels were reduced in both the mania and depression subgroups (mania: 67.19 ± 21.52 ng/ml, depression: 63.43 ± 20.67 ng/ml; *P* < 0.001), while no significant difference was observed between two groups (*P* > 0.05). Spearman correlation analyses revealed that the levels of serum IGF-2 were negatively correlated with the YMRS scores in BD patients (r = -0.522, *P* < 0.001). Furthermore, IGF-2 was found to be an independent contributor to the severity of symptoms in patients with manic episodes (*B* = -0.610, t = -5.299, *P* < 0.001).

**Conclusion:**

Lower serum IGF-2 levels were found in BD patients and correlated with the severity of the manic symptoms in these patients during manic episodes. These results suggest that reduced IGF-2 levels might be involved in the pathophysiology of BD, and serum IGF-2 could be a peripheral biomarker for the evaluation of the severity of manic symptoms in BD patients.

## 1 Introduction

Bipolar disorder (BD) is a chronic, serious mood disorder that is characterized by recurrent mood episodes that depressive episodes alternating with mania and/or hypomania or only manic episodes (1). It usually manifests in adolescents and has a significant impact on the social function of patients. Bipolar disorder has one of the highest rates of disability in the world, placing sixth among the top 10 medical conditions that cause disability in adults between the ages of 15 and 44 (2). Although the use of mood stabilizers such as lithium or valproate can help manage symptoms, prevent mood episodes or reduce the severity in most patients with BD, there are still some patients with poor therapeutic efficacy. Therefore, a thorough study of the pathological mechanisms of BD will be helpful to the treatment of this disorder.

Accumulating evidence has indicated that neurodevelopmental defects may underlie the pathophysiology of BD (3). For example, Soares and Mann reported that individuals with BD have a larger third ventricle and cerebellum compared to normal controls (4). A study using the neuroimaging technique of diffusion tensor imaging (DTI), which is used to assess connectivity between various regions proximal and distal to BD (5), revealed that patients with BD had consistently declining fractional anisotropy values in the corpus callosum and frontal limbic tract (6). Circle RNAs (circRNAs) are highly expressed in the brain, and most of these circRNAs are extremely active at neuronal synapses (7). Compared to normal controls, a significant dysregulation of 55 circRNAs with a bias towards downregulation was found in BD patients (8).

Neurotrophic factors, also referred to as neurotrophins, are growth factors originally identified in the nervous system. As indicated by the name, neurotrophic factors are essential for the survival and development of neurons. Insulin-like growth factors (IGFs) are a family of neurotrophic factors that play an important role in the cell cycle, including IGF-1, IGF-2, insulin-like growth factor binding protein (IGFBP), and so on. They affect cell survival in adult tissues and promote cell growth, survival, migration, and differentiation (9). Abnormality in IGF-1 signaling is suggested to be implicated with the aetiology of BD (10). Accordingly, patients with BD had lower IGF-1 mRNA expression in the subependymal zone, which may have a potential impact on neurogenesis in this disorder (11). IGF-1 levels in the peripheral blood of patients with BD were significantly increased in comparison with the control individuals (12). Lithium is the mainstay in the treatment of BD. A genome-wide expression analysis of lymphoblastoid cell lines (LCLs) revealed that IGF-1 was significantly overexpressed in lithium-responsive BD patients compared to non-responders patients (13). IGF-2 is a type of neurotrophic factors, playing an essential role in cell differentiation and survival, neuronal development, and synaptic plasticity by binding and activating the corresponding receptors (14, 15). IGF-2 is abundantly expressed during fetal development and has a major effect on embryonic growth (16). Studies of mice have reported that knockout of Igf2 results in growth retardation, whereas overexpression of Igf2 results in overgrowth (17). Deficiency of IGF-2 expression was shown to contribute to the growth restriction in patients with the Silver-Russell syndrome, a syndromic growth-retardation disorder (18). In the adult brain, IGF-2 that is generated from myelin sheaths, choroid plexus, leptomeninges and hypothalamus, can affect cognition, memory and emotion by regulating the function of neurons (19, 20). Moreover, the role of IGF-2 in the brain is also sustained by data showing its alterations as a common feature across a variety of psychiatric and neurological disorders, including schizophrenia, Alzheimer’s disease, depression, and so on (21) Pai et al. showed that there was a prominent hypomethylation of an enhancer within the IGF-2 gene in neurons from the prefrontal cortex of patients with BD, indicating that the epigenetic activity of IGF-2 enhancers may increase the synthesis of dopamine associated with major psychosis (22). However, whether IGF-2 signaling is implicated in the pathophysiological processes of BD is unknown.

In the present study, we aim to investigate the role of IGF-2 signaling in the pathophysiology of BD by studying whether (1) serum IGF-2 was changed in Han Chinese patients with BD and (2) there was any association of serum IGF-2 levels with the psychopathological symptoms in these patients.

## 2 Methods

### 2.1 Subjects

We used Power Analysis and Sample Size (PASS) software to calculate the sample size. It suggests a minimum sample size of 17 in the subgroups and a minimum sample size of 51 in total. Considering the need for a clinic study, a sample size of 125 were selected in the study. 78 patients with BD, including 23 patients who diagnosed acute depressive episode and 55 patients who diagnosed acute manic episode, were recruited from Jiangxi Mental Hospital. The diagnosis of BD was made using DSM-IV criteria and was carried out after a structured clinical interview (Mini-International Neuropsychiatric Interview, Mini-plus). Two psychiatrists confirmed their BD diagnosis. The exclusion criteria included the following: additional axis I and II DSM-IV diagnoses, comorbidities, allergic and autoimmune diseases, current pregnancy, and other physical disorders, such as cardiac and cerebral infarction, in the past 3 months. All the recruited patients were drug naive or had stopped taking any mood stabilizer or antipsychotic for at least 3 months prior to taking part in the study. 50 healthy controls who matched the patients by age, gender, and body mass index (BMI) were recruited from the local community. All the subjects were from Jiangxi Province, China. They have the same balance diet and their lifestyles are similar. Structured clinical interview was carried out in healthy controls and individuals with personal or family history of mental disorders were excluded in this study. All participants were Han Chinese ethnicity. None of them suffered from substance abuse or substance dependence, and none were taking immunosuppressants.

The Young Mania Rating Scale (YMRS) and the Hamilton Depression Rating Scale, 17-item version (HAMD-17), were used to confirm whether the patients were in a manic or a depressive episode, respectively. Patients with YMRS > 20 or HAMD-17 > 7 were included in the study. To better understand the association of serum IGF-2 with the severity of depressive or manic symptoms, we did not include mixed episodes (both YMRS > 20 and HAMD-17 > 7 points) in this study.

The research was approved by the Institutional Review Board at Jiangxi Mental Hospital and was also conducted in accordance with the Declaration of Helsinki. We obtained written informed consent from each participant or his or her legal guardians.

### 2.2 Sample collection and measurement of serum IGF-2 content

Following overnight fasting, blood samples were obtained from a forearm vein between 7:00 and 8:00 a.m. The serum was separated from the samples and then stored at -80°C before laboratory measurement. The serum level of IGF-2 was determined using an enzyme-linked immunosorbent assay (ELISA) with commercially available kits (Wuhan USCN Business, Wuhan, China). In this type of ELISA, the antigen is bound to the polystyrene microtiter plate first. The antiserum containing the anti-peptide antibody is then added to the well and allowed to bind. Finally, a second antibody, specific for the first antibody and labeled for detection, is added to the well and allowed to bind. The second antibody has an enzyme conjugated to it. This enzyme catalyzes the formation of colored substance. This colored substance is then quantified and the amount of antibody present can be calculated. This IGF-2 kit can specifically detect IGF-2 and has no obvious cross-reaction with other similar substances. The sensitivity of this IGF-2 assay was 0.263 ng/ml, and the inter- and intra-assay coefficients of variation were 10% and 12%, respectively. All participants’ samples were processed together in the same assay batches. Each sample was assayed in duplicate.

### 2.3 Statistical analysis

The Statistical Product and Service Solutions (SPSS) 26.0 software was used to analyze the data. For descriptive information detailing demographic and clinical features, the data were presented as mean ± standard deviation (SD). Because none of the groups passed the Shapiro-Smirnov test and the Quantile-Quantile Plot indicated that all the data was not normally distributed, non-parametric tests were adopted for data analysis. Dichotomous data, such as gender, between the control and patient groups were compared using chi-square testing. The Kolmogorov-Smirnov and Mann-Whitney U tests were chosen for comparison between groups. The results of the Kolmogorov-Smirnov test showed that serum IGF-2 levels were significantly lower in both the mania and depression subgroups compared to healthy controls. *Post hoc* comparisons were conducted using Bonferroni’s test to identify the differences in serum IGF-2 levels between the two subgroups. Considering the non-normally distributed data, Spearman analysis was used to identify the correlation between variables and serum IGF-2 levels. Additionally, in order to exclude other factors, Partial correlation analysis was performed to confirm the correlation between YMRS scores and serum IGF-2 levels. Finally, a multivariate regression analysis was conducted to determine the independent factors influencing YMRS scores. A significance threshold was set at *P* < 0.05.

## 3 Results

50 healthy controls (26 male, 24 female), 55 BD patients with acute manic episode (21 male, 34 female), and 23 BD patients with acute depression episode (10 male, 13 female), were enrolled in this study. The clinical and demographic characteristics of the healthy controls and BD patients are shown in **Table 1**. There was no difference in age, gender, and BMI between the healthy control and BD patient groups (*P* > 0.05). For BD patients, no difference was observed in illness duration or times of episode between the mania and depression subgroups (*P* > 0.05). However, the age of first onset in depression subgroup was lower than that in mania subgroup (*P* < 0.01). Furthermore, there was a trend toward a lower age in depression subgroup in comparison with the manic subgroup, but no significant difference was found (*P* > 0.05).

**Table 1:** Demographic and clinical features of controls and BD patients enrolled in this study.

Variables	Controls (n=50)		BD patients (n=78)				*P* value
			Mania (n=55)		Depression (n=23)		
	M	SD	M	SD	M	SD	
Age (years)	29.28	3.83	30.49	8.44	24.56	7.24	0.098^a^
BMI (kg/m^2^)	23.03	2.72	22.48	3.44	22.59	3.45	0.208^a^
Illness duration (years)	–	–	7.72	6.29	5.20	4.07	0.117^a^
First onset age (years)	–	–	22.98	6.00	17.73	5.71	**<0.001** ^a^
Times of episode	–	–	3.49	1.79	3.17	1.74	0.358^a^
YMRS score	–	–	35.34	6.78	–	–	
HAMD-17 score	–	–	–	–	25.17	5.6	
IGF-2 level (ng/ml)	88.72	31.55	67.19	21.52	63.43	20.67	**<0.001** ^b^

^a^Mann-Whitney test (healthy control group compared with BD group, or difference between the manic and depressive subgroups).

^b^Kruskal-Wallis test (healthy controls, manic episode subgroup and depressive episode subgroup compared with each other).

The bold values mean that the p < 0.05.

**Figure 1:**
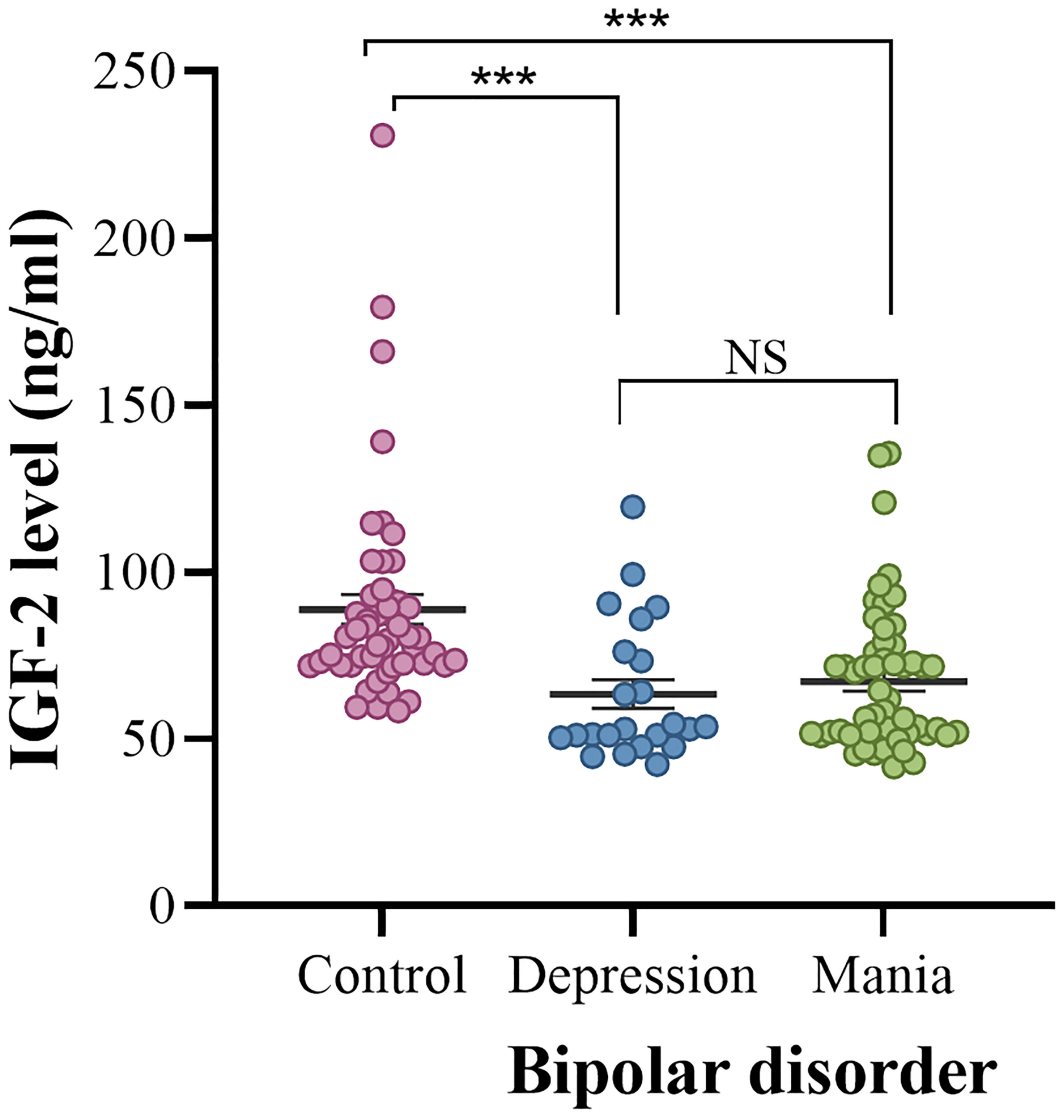
Serum IGF-2 levels in the controls and the depression and mania subgroups. The sample means are indicated by the black bars. ***indicates P < 0.001. NS, no significance.

In comparison to healthy controls, serum IGF-2 levels were significantly lower in patients with BD (88.72 ± 31.55 vs. 66.08 ± 21.22 ng/ml; z = -5.402, *P* < 0.001). BD patients were divided into manic episode and depressive episode subgroups. We found that serum IGF-2 levels were much lower in both the mania and depression subgroups than those in healthy controls (mania: 67.19 ± 21.52 ng/ml, depression: 63.43 ± 20.67 ng/ml, controls: 88.72 ± 31.55 ng/ml; H = 29.692, *P* < 0.001) (**Figure 1**). *Post hoc* comparisons using Bonferroni’s test showed that there was no significant difference in the levels of serum IGF-2 between the mania and depression subgroups (*P* > 0.05). Furthermore, there was no significant difference in serum IGF-2 level between males and females in these groups (*P* > 0.05).

**Figure 2:**
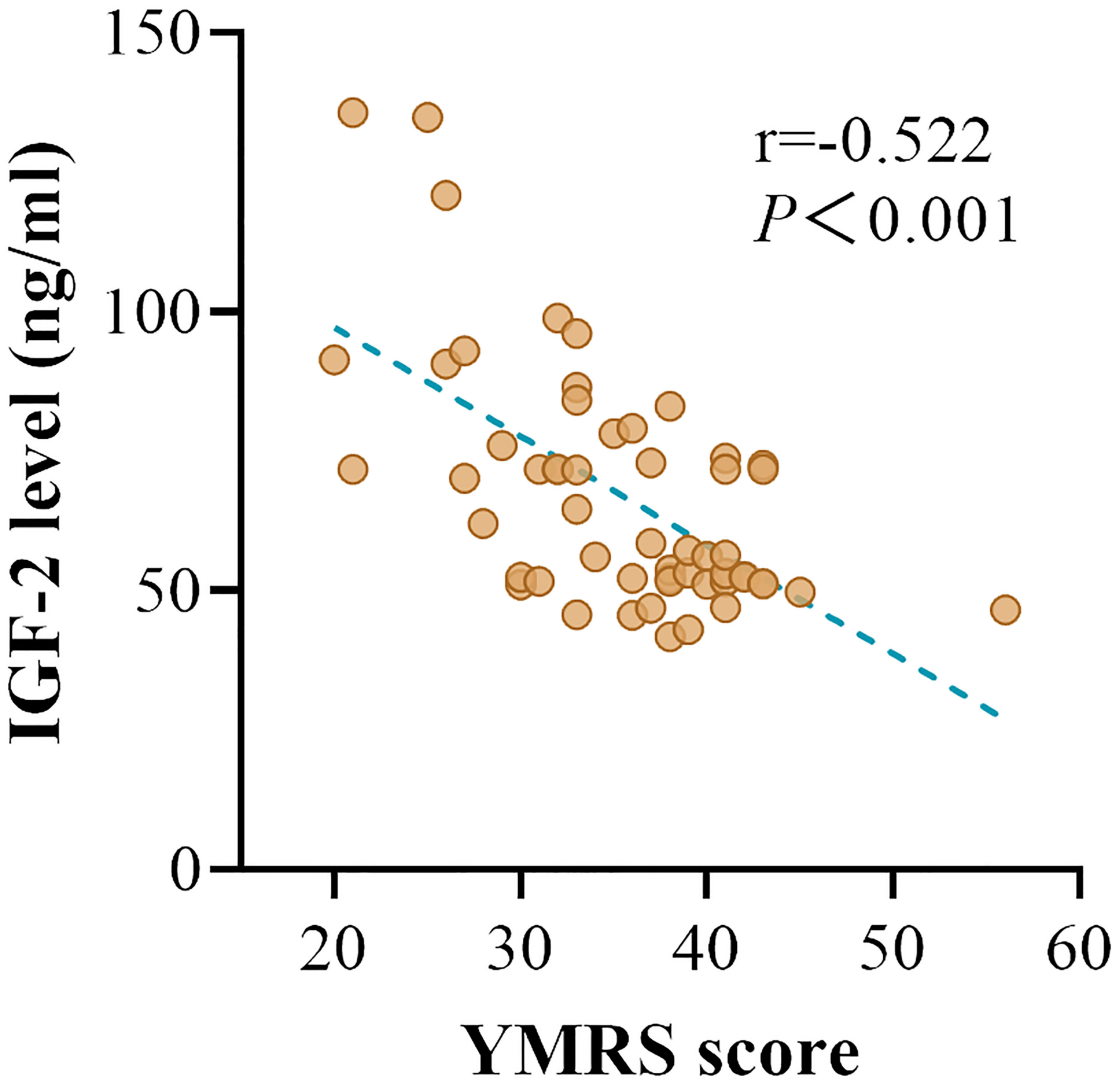
The correlation between the serum IGF-2 levels and YMRS scores in BD patients with manic episode.

In patients with manic episode subgroup, we found a negative association between serum IGF-2 levels and YMRS scores (r = -0.522, *P* < 0.001) (**Figure 2**). In patients with depressive episode subgroup, no relationship was found between serum IGF-2 levels and HAMD-17 scores (*P* > 0.05). In addition, serum IGF-2 levels had no correlation with the variables including gender, age, age of onset, duration of illness, BMI, or number of episodes in BD patients (*P* > 0.05). Partial correlation analysis revealed that the correlation between IGF-2 levels and YMRS scores still existed in patients with manic episode when controlling for clinical variables including gender, age, age of onset, illness duration, and BMI (r = -0.612, *P* < 0.001). Finally, we conducted a multivariate regression analysis to reveal the independent factors influencing YMRS scores and found that serum IGF-2 levels were one of those factors (*B* = -0.610, t = -5.299, *P* < 0.001).

## 4 Discussion

The present study demonstrates that patients with BD had lower serum IGF-2 levels in comparison to healthy controls, and serum IGF-2 levels were negatively correlated with the severity of manic symptoms in patients with manic episodes. To our knowledge, this is the first study to report the association of serum IGF-2 levels with the manic and depressive symptom severity in BD patients.

The growth and development of neural cells is affected by a variety of neurotrophic factors, cell membrane receptors, and peptide hormones. IGF-2 is extensively expressed in the brain during the development period and adulthood, and it can affect cell differentiation and survival, neuronal development, and synaptic plasticity. In this study, we found that serum IGF-2 levels were significantly lower in patients with BD than in the healthy control group. Given the importance of IGF-2 signaling in regulating neurodevelopment and emotion, our result not only provides evidence for the neurodevelopmental hypothesis of BD but also reveals that IGF-2 might be involved in the pathophysiological process of this disorder. It is worth mentioning that the change in serum IGF-2 in BD patients could be attributed to the illness itself or other confounding factors, such as the use of mood stabilizers or antipsychotics. In the current study, we recruited patients who had stopped taking any mood stabilizer or antipsychotic for at least 3 months before entering this study. Moreover, a prominent hypomethylation of an enhancer within the IGF-2 gene was found in neurons from the prefrontal cortex of patients with BD (22). Thus, in combination with the finding that reduced serum IGF-2 level had a relationship with the severity of manic symptoms, we postulate that the change in IGF-2 expression is more likely to ascribe to the disease *per se*, rather than a phenomenon secondary to mood stabilizer or antipsychotic treatment. However, this assumption still needs to be confirmed by detecting IGF-2 levels in first-episode and drug-naive patients with BD. Nevertheless, the fact that change of IGF-2 signaling in BD supports the neurodevelopmental hypothesis of this disorder, and provides more information about the pathophysiology of BD and might have potential clinical implications for the development of biomarkers for the symptom evaluation in the future.

The YMRS and HAMD-17 are the most widely used scales for evaluating manic and depressive symptoms in BD associated studies. BD patients were divided into mania and depression subgroups according to the scores of the YMRS and HAMD-17 scales in this study. We found that serum IGF-2 levels were significantly lower in both the mania and depression subgroups when compared to healthy individuals. No significant difference was found in the levels of serum IGF-2 between the manic and depressive patients. These data reveal that reduced levels of serum IGF-2 are a disease characteristic of BD. Correlation analysis showed that there was a significantly negative correlation between serum IGF-2 levels and the YMRS scores in BD patients with acute manic episode. Partial correlation analysis demonstrated that the correlation between IGF-2 levels and YMRS scores still existed when controlling for age, gender, age of onset, illness duration, and BMI. Multivariate linear regression analysis revealed that serum IGF-2 level was an independent factor influencing the YMRS score. These results suggest that BD patients with lower IGF-2 levels would be more likely to have severer manic performances. However, although a correlation between IGF-2 levels and manic symptoms in BD patients is observed, it should be noted that this does not imply causation. The relationship between serum IGF-2 level and manic symptom severity may be a reverse causality and there may be some other underlying factors that might influence both IGF-2 levels and symptom severity. More studies are needed to disclose the role of IGF-2 signaling in the pathophysiology of BD.

Previous studies have demonstrated a role for IGF-2 signaling in major depressive disorders (MDD) (23). Fernández-Pereira et al. reported that the levels of plasma IGF-2 were significantly increased in patients with MDD and tended to normalize after antidepressant treatment (24). The gene expression of *Igf2* was found to be downregulated in the hippocampus in a rat model of depression (25), and intrahippocampal injection of IGF-2 could mitigate depressive-like behaviors in both rats and mice models (26, 27). In addition, variable methylation of the *Igf2* gene has also been found to be related to the clinical manifestation of depression in monozygotic twins (28). In the current study, we found that serum IGF-2 levels were significantly lower in BD patients with depressive episode, which is consistent with the change in MDD, while no association of IGF-2 levels with HAMD-17 scores was found in these patients. These results not only suggest that IGF-2 signaling has different roles in the pathophysiology of BD and MDD, but also provide evidence for the difference in the pathogenesis of BD and MDD. Indeed, although MDD and BD share some clinical features and genetic risk factors (29), the pathophysiological mechanisms of these two disorders are largely different. For example, distinct gut microbial compositions were identified in MDD patients compared to BD patients (30). Homocysteine levels were elevated in MDD and BD patients, and were higher in BD patients than in MDD patients (31). The resting state functional magnetic resonance imaging (fMRI) studies revealed that BD patients had a specific increased amplitude of low frequency fluctuations in the right brain regions and bilateral cerebellum (32, 33), while specific abnormalities were found in functional connectivity in the amygdala-ventral PFC in MDD (33, 34). Unlike MDD, our present study found that lower serum IGF-2 levels were not correlated with the severity of depressive symptoms in BD patients with depressive episode. However, it could not rule out the possibility that no association between depressive symptom severity and serum IGF2 is due to small sample size or other confounding factors in this study. Thus, this preliminary conclusion needs to be verified by further experiments.

Lower IGF-2 level is correlated with the severity of the manic symptom in BD patients, indicating IGF-2 signaling is involved in the pathophysiological process of BD. However, how does IGF-2 affect mania is not clear. It is reported that dopamine imbalance is critical to the pathogenesis of major psychosis (35). Abnormalities in DNA methylation in an enhancer within the IGF-2 gene have been found in the brains of patients with BD (22, 36). Furthermore, hypomethylation of the IGF-2 enhancer can increase tyrosine hydroxylase (TH) protein levels, which can affect the synthesis of dopamine levels (22). Thus, downregulation of IGF-2 signaling may be one of the contributing factors to a higher dopamine level in patients with BD. Chronic stress and inflammation are the primary biological mechanisms behind bipolar disorder (37). The pro-inflammatory cytokine interleukin 6 (IL-6) can activate serotonin- and tryptophan-degrading enzymes or have an effect on cholinergic and muscarinic substances to induce mania (38–40). IGF-2 can activate the Akt-NF-κB pathway to regulate IL-6 expression (41, 42). Therefore, a change in neuroinflammation caused by abnormal IGF-2 expression might also be a triggering factor for the manic symptoms in patients with BD. Further studies that use animal models are required to elucidate the mechanism of IGF-2 signaling in the pathogenesis of BD.

There are some limitations in this study. First, the sample size is small, and all the subjects were recruited from the same institution. This poses potential limitations to the generalizability of our findings. A larger number of subjects who are recruited from different institutions are needed to confirm this conclusion. Second, we measured IGF-2 levels in serum but not in the brain tissue or cerebral spinal fluid (CSF) of patients. It is not clear whether IGF-2 levels in peripheral change are parallel with the levels in the brain. Third, this is a cross-sectional study and there is a potential selection bias. A causal relationship between abnormal IGF-2 signaling and the pathogenesis of BD could not be drawn out in this study. More studies are needed to disclose the role of IGF-2 signaling in the pathophysiology of BD. Fourth, family socioeconomic status (SES) has been shown to be associated with depressive symptoms in children and adolescents (43). However, we did not consider SES as a demographic variable in this study, which might limit the generalizability of the results. Additionally, although an association between lower serum IGF-2 and the severity of manic symptoms in patients with BD was found in this study, the mechanisms through which IGF-2 affects manic performances need to be investigated.

In conclusion, our present study reveals that serum IGF-2 levels were significantly lower in patients with BD and that lower IGF-2 levels were correlated to the severity of manic symptoms in these patients. Although a causal correlation between serum IGF-2 levels and manic symptoms in BD patients could not be drawn out, the fact that change of IGF-2 signaling in BD supports the neurodevelopmental hypothesis of this disorder and provides more information about the potential pathophysiological mechanisms of BD.

## Data availability statement

The raw data supporting the conclusions of this article will be made available by the authors, without undue reservation.

## Ethics statement

The studies involving humans were approved by The Institutional Review Board at Jiangxi Mental Hospital. The studies were conducted in accordance with the local legislation and institutional requirements. The participants provided their written informed consent to participate in this study.

## Author contributions

SY: Writing – original draft, Investigation, Methodology, Software. YZ: Writing – review & editing, Data curation, Funding acquisition, Investigation. ZL: Writing – review & editing, Project administration. CL: Writing – review & editing, Data curation, Investigation. JX: Writing – review & editing, Investigation. JZ: Writing – review & editing, Data curation, Software, Supervision. YL: Writing – review & editing, Investigation. BW: Writing – review & editing, Supervision. CC: Writing – review & editing, Funding acquisition, Resources, Supervision. YY: Writing – original draft, Writing – review & editing, Data curation, Funding acquisition, Resources, Supervision.
