# Corrigendum: Lower serum insulin-like growth factor 2 level in patients with bipolar disorder is associated with the severity of manic symptoms during manic episodes

**DOI:** 10.3389/fpsyt.2024.1408899

**Published:** 2024-04-12

**Authors:** Shi-Yi Ye, Ying Zhao, Zhao-Bo Liu, Cui-Pin Luo, Jian-Wen Xiong, Jin-Qiong Zhan, Yi-Heng Li, Bo Wei, Chun-Nuan Chen, Yuan-Jian Yang

**Affiliations:** ^1^ Department of Psychiatry and Biological Psychiatry Laboratory, Jiangxi Mental Hospital & Affiliated Mental Hospital, Jiangxi Medical College, Nanchang University, Nanchang, Jiangxi, China; ^2^ The 3^rd^ Clinical Medical College, Jiangxi Medical College, Nanchang University, Nanchang, Jiangxi, China; ^3^ Department of Pharmacy, Union Hospital, Tongji Medical College, Huazhong University of Science and Technology, Wuhan, China; ^4^ Department of Psychiatry, Third People’s Hospital of Ji′an City, Ji′an, China; ^5^ Nanchang City Key Laboratory of Biological Psychiatry, Jiangxi Provincial Clinical Research Center on Mental Disorders, Jiangxi Mental Hospital, Nanchang, Jiangxi, China; ^6^ Department of Neurology, The Second Clinical Medical College, The Second Affiliated Hospital, Fujian Medical University, Quanzhou, China

**Keywords:** bipolar disorder, insulin-like growth factor-2, manic symptoms, severity, serum

In the published article, an author name was incorrectly written as Yin Zhao. The correct spelling is Ying Zhao.

The authors apologize for this error and state that this does not change the scientific conclusions of the article in any way. The original article has been updated.

